# Mechanisms of Gravitational Sensitivity of Osteogenic Precursor Cells

**Published:** 2010-04

**Authors:** L.B. Buravkova, P.M. Gershovich, J.G. Gershovich, A.I. Grigorʹev

**Affiliations:** State Scientific Centre of Russian Federation - Institute for Biomedical Problems Russian Academy of Sciences

**Keywords:** osteopenia, gravitational sensitivity of cells, microgravity, osteoblasts, osteogenic precursor cells, multipotent mesenchymal stromal cells, differentiation, cytoskeleton

## Abstract

This report is a detailed review of the current data on the mechanic and gravitational sensitivity of osteoblasts and osteogenic precursor cells *in vitro*. It summarizes the numerous responses of cells with an osteoblastic phenotype and osteogenic precursor cells and especially their responses to the alteration of their mechanic or gravitational surroundings. The review also discusses the osteogenic cell’s pathways of signal transduction and the mechanisms of gravitational sensitivity. It was shown that the earliest multipotent stromal precursor cells of an adult organism’s bone marrow can sense changes of intensity in a gravitational or mechanic field in model conditions, which may play a certain role in the development of osteopenia in microgravity.

## INTRODUCTION

During the evolution, the skeletal system of land vertebrates adapted itself to an environment in which one of the most prominent and constant factors is gravity. This factor has determined the morphogenesis and structure of all land animals. Certain elements of the skeleton have evolved for maintaining posture and achieving active locomotion and thus are constantly experiencing static and dynamic strain as a result of “defying the gravitational force.” Since humans have started exploring the outer space, the effect of microgravity on the skeletal system has become an important issue, as a lack of mechanic stress (microgravity, hypokinesia, hypodynamia, immobilization) can lead to the loss of bone mass caused by insufficient mechanic impulses and gravity–induced deformations, which are not capable of supporting the integrity of the skeletal remodeling processes [1, 2].

Studies conducted in the last decade have conclusively demonstrated that cultured cells of osteoblast phenotype are sensitive to microgravity [3–8]. However, one question remains unanswered: How can microgravity affect the numerous functional aspects of less mature cell forms, namely the progenitor cells?

During post–natal development the main source of precursor cells is the bone marrow, which is closely connected with bone tissue both in formation and in functioning. Among the numerous components of the bone marrow stroma, there is a minor population of cells which is localized in the perivascular region of the marrow but differs from endothelial or smooth muscle cell populations by the expression of several surface antigens and by the cell’s ability to differentiate into tissue cells of mesenchymal origin. So these cells posses all the characterstics of multipotent mesenchymal stem/stromal cells (MMSC) [9, 10]. MMSC were first isolated from animal bone marrow in the 70’s of the 20^th^ century by A.Y. Friedenstein and his collegues. Later, MMSC were found and extracted from human bone marrow. A large number of studies showed that * in vitro * MMSC can differentiate into the cellular elements of bone, cartilage and fatty tissues, as well as support and regulate hematopoiesis [11–13]. It is well known that osteoblasts of different stages of maturity have different degrees of gravitational sense [14, 15], however, the mechanisms of gravitational sensitivity of less committed cells of the bone tissue have only recently started to be elucidated.

## Possible mechanisms of the gravity effect on the cellular level

Comparison of the results obtained in * in vitro * experiments, with the changes that take place in a human organism under the influence of microgravity, provides an opportunity to differentiate and establish the role of cellular reactions in forming physiological responses, since it allows to factor out the effects of the integral regulating systems of the human organism. The development of the views on cellular gravitational sensitivity per se can be seen in a series of reports [16–20]. Discussions of whether an *in vitro * single cell or a cell population can sense changes in the gravitational field are still very heated. Despite this, an enormous body of experimental data undoubtedly indicates that several types of cultured cells are sensitive to gravity. In particular, it was demonstrated that microgravity causes multiple and often reversible morpho–functional alterations, including remodeling of the cytoskeleton, change of gene expression and a mosaic rearrangement of the intracellular regulatory machinery. These alterations are reviewed in detail in [5, 19, 21, 22].

It seems that undifferentiated mammalian cells do indeed have structural elements that may play the role of “gravitational sensor” and “sense” the intensity of a mechanical tension, and that many intracellular processes can depend on the value of the gravitational force. The most probable candidates for the role of these structures are various elements of the cytoskeleton, the nucleus, intracellular organelles and also certain cell surface receptors (integrins), which interact both with cytoskeletal structures and the extracellular matrix. These structures are able to sense strains and deformations in the matrix which are caused either by a gravitational or mechanical field and transfer this signal to intracellular messengers, which then cause a cellular response to the gravity changes [18, 23, 24]. Based on several theoretical considerations and practical observations, it is supposed that the gravitational sensitivity of the cells which grow on a surface is a function dependant on two variable parameters: The level of cell adhesion to the substrate and the strength of the intercellular interactions, while the realization of these interactions is in direct proportion to the amount of invested energy [17]. The indirect effect of microgravity at the cellular level can manifest itself in changes of the physico–chemical parameters of the medium, especially the processes of convection, sedimentation and also concentration gradients, which are all gravity–dependant and can thus be altered in microgravity [20, 25].

## Mechanic and gravitational sensitivity of various types of bone tissue cells: effects on the proliferative potential of cells

For a long time, osteocytes and the mature inactive osteoblasts were widely accepted to be the most likely candidates for a mechanosensor in the bone tissue [14, 15]. It was supposed that this process was performed via cell–cell junctions, formed by integrins, which interact with elements of the actin cytoskeleton (actin, vinculin, etc.) inside the cell and with various proteins of the bone matrix outside the cell, thus forming a continuous network which encompasses osteocytes and the bone matrix. It was thought that this ever–present and all–encompassing structure could sense and potentiate the effect of even miniscule mechanical stimuli [26].

It was demonstrated on bone cell cultures that certain types of mechanic stimulation, such as pulsatile fluid flow or mechanic strain, can trigger a cascade of regulatory reactions. The latter included a transient increase in the production of low molecular weight messengers, such as NO, expression of the inducible prostaglandin synthase (Cox–2) and secretion of porstaglandins (PGE_2_, PGI_2_), which were involved in the increase of the intracellular calcium concentration, in the activation of the inositol–3–phosphate signal cascade [27], and in increasing cAMP and IGF–I levels, activation of proliferative and differentiation processes in bone cells [15], and activation of cytoskeletal remodeling [28]. Nevertheless, effects from different types of mechanic stimulation are not identical [29, 30], and cells at different stages of maturity can react to the same mechanical stimulus either in the same manner [28], or differently [14, 15]. Such selectiveness and variability of the bone cell responses towards various types of stimuli seems to be caused by the unalike distribution of differentiating and mature cells within * in* *situ * bone tissue, as well as by the differences in their maturity and their functions.

It is well known that the proliferative activity of osteoblasts is controlled by a wide range of bioactive compounds, as well as by mechanical signals. In particular, it was shown that Cox–2 expression and PGE_2_ production increase in osteoblasts in response to the growth factor TGF– β and that this effect is required for the transition between the G1–phase and the S–phase, DNA replication and active proliferation [5]. Notably, different types of mechanic stiumuli, as well as hypergravity [31], can increase PGE_2_ production, which implicates PGE_2 _in the anabolic effects of mechanical stress. Surprisingly, the studies conducted in microgravity detected both an increase in PGE_2_ production and also a decrease of Cox–2 mRNA levels and PGE_2_ production in conjunction with an overall decrease of cell growth under microgravity [5]. The latter effect was accompanied by alterations in the structure of the actin cytoskeleton.

Studies that analyze the effect of mechanical stress on progenitor cells are of special interest. It was determined that human MMSC express both Cox–1, and Cox–2, and produce PGE_2_ at a higher level than osteoblast–like cells derived from them. It was also found that the increased production of this metabolite in MMSC was associated with an increase in the expression of a membrane–bound prostaglandin–synthase. Also endogenous MMSC PGE_2_ production controls the synthesis of the osteogenic growth factor BMP–2 [32]. It seems that MMSC, as well as mature osteoblasts and osteocytes, can be thought of as mechanosensory bone tissue cells, since anisotropic single axis mechanic deformation of MMSC cultured on special elastic membranes causes overall changes in the gene expression pattern, lowers the activity of certain signal transduction pathways (Jagged1), **** and activates cell proliferation [33]. Thus, the view that has dominated for some time now, that bone tissue cells with low–level differentiation cannot or can hardly sense mechanic stimuli, must obviously be corrected. It is worth noting that the data which show changes in the proliferative activity of cells with osteoblastic phenotype under altered gravity are fairly controversial. Inhibition of osteoblast cell proliferation has been shown both in microgravity and in experiments that modeled these conditions [6, 34, 35]. On the other hand, the use of Random Positioning Machine (RPM) did not inhibit 2T3 mouse preosteoblast growth [36]. The proliferative activity of MMSC during osteogenic differentiation did not change in a rotational bioreactor [37], decreased after incubation in a clinostat [38], and actually even increased after cultivation in a 3D–clinostat [39].

It seems that the most probable effect of microgravity on the osteogenic precursor cells is a change in the normal cell response to the anabolic influence of growth factors. Currently many researchers are of the opinion that the observed cell reactions are not caused by physical loss of growth factor receptors (for instance EGF, PDGF), but more likely by a change in the signal transduction system caused by microgravity [5, 40]. This opinion has led scientists into thorough research of candidate intracellular mechanisms and signaling pathways. According to modern views, the major routes of all three main directions of MMSC differentiation include the activation/repression of MAP–kinase cascades (mitogen–activated proteinkinases) [41]. It was demonstrated that the activation of the well–known MAP–kinase cascade (ERK1/2) is mainly achieved through a Ras–dependant signaling pathway which is activated in response to binding of growth factors with their receptors [41]. It is supposed that growth factors such as BMP–2 and IGF–I, cause their positive mitotic effect on MMSC via the activation of the MAP–kinase cascade. This process also includes proteinkinase D, but not protein kinase С [42]. Notably, the increase in MMSC proliferation observed under the effect of pulsatile fluid flow is also realized through the calcium signaling system and the MAP–kinase cascade, which indicates the existence of a general mechanism for transforming mechanical signals into biochemical ones in osteogenic precursor cells of varying degrees of maturity [43].

## The role of adhesion receptors in regulating precursor cell functions and in sensing mechanical and gravitational stimuli.

The question of whether the immunophenotype of precursor cells remains intact under conditions of altered gravity may be of much importance for several reasons. First, the main CD–clusters, which are expressed on the MMSC membrane, regulate various aspects of precursor cell functioning. Since they are surface receptors for growth factors and thus mediate the interactions between MMSC and hemopoetic precursors and lymphocytes, they modulate the maturation and activity of the latter and take part in the interaction of cells with molecules of the extracellular matrix [11, 13, 44]. Second, the role of some antigens in the realization of unique stem cell differentiation potentials is still unknown. Instances of the effect of model microgravity on the expression of specific MMSC surface markers are rare and controversial. Specifically, one study determined that a 7–day incubation in a 3D– с linostat caused an increase of the population ratio of human MMSC cells expressing stromal cell antigens CD44+, CD90+, CD29+ [39]. Another study showed that a 6–day incubation in a horizontal с linostat decreased the number of cells bearing the CD105 and HLA A,B,C antigens in a culture of human bone marrow MMSC [45]. Our own studies show that a 5–day incubation of MMSC on a RPM causes an increase in the number of cells expressing integrin CD49b, but does not affect the percentage of cells, expressing CD29 [46].

Perhaps the most interesting aspect of the biological peculiarities related to osteogenic precursor cell immunophenotype is the potential role of certain antigens in the mechanisms of mechanic and gravitational sensitivity. The mechanochemical hypothesis proposes that integrins and other receptors on the cell surface play an important role in the physical interaction between the extracellular matrix and the cytoskeleton and in sensing gravitational signals [23, 24]. A complex study, which looked into the molecular functions of integrins, demonstrated that simple clusterization of integrins on the cell surface in response to signals from the extracellular matrix triggers the transmembrane activation of 20 major mediators of signal transduction, including cytoskeleton effector proteins RhoA, Rac, Ras, Raf, and MAP–kinases MEK, ERK and JNK. Notably, the use of cytochalasin D and tyrosine kinase inhibitors did not abolish the aggregation of integrins with FAK and cytoskeleton proteins (vinculin, talin and α –actinin) [47].

The potential role of several mentioned antigens in the response of bone cells to a rapid decrease in the mechanical stress level is very intriguing. Proof of the fact that integrins (namely, β 1–integrin or CD29) play a role in osteoblast mechanical signal sensitivity was obtained in a study conducted on mice, which expressed β 1–integrin in the normal amount, and transgenic animals, which had a dominant negative * β* *1–integrin * gene introduced into their genome. Adult mice at 2 months of age exhibited an osteopenic phenotype, displayed a characteristic decrease in the bone tissue mass of the hind limbs, and also decreased durability and robustness of the tissue, despite a normal level of bone remodeling [48]. Another study showed activated expression of the alpha2–integrin during the course of MMSC differentiation in conditions of simulated microgravity [49]. Recent studies in mechanobiology focus not only on integrins, but on other receptors of cell adhesion as well, especially CD44 (HCAM). A study on a MC3T3–E1 osteoblast culture showed that mechanic stress caused by pulsatile fluid flow led to an increase in the level of osteopontin mRNA. This protein is a major component of the bone tissue matrix and is a ligand of CD44 [50]. Mice lacking the *OPN * gene exhibit resistance to unloading by tail–suspension and lose less bone tissue mass than the wild–type mice. Bone marrow MMSC from suspended *OPN * –negative mice, cultivated * ex vivo * , are characterized by a normal ability to form mineralized nodules, as compared to decreased mineralization in cultures extracted from wild–type mice after they were suspended [51]. In connection with this, it is interesting that a 5–day incubation of rat osteoblasts under microgravity led to a decrease in osteopontin expression levels, but increased the expression of CD44, while the expression level of β 1–integrin (CD29) remained constant [7].

It is known that CD44 plays a role in binding and regulating matrix metalloproteinases (MPPs) [52]. MMSC express and produce various types of MPPs (–2, 3, 10, 11, 13, 14), and mechanical stress causes increased activity of these enzymes, and of collagenase (MPP–13) in particular, and interestingly, this increase takes place on the post–translational level [53]. These studies demonstrate that a change in the specific balance of collagenase activity level or expression can play a distinct role in the mechanisms of collagen matrix maturation and destruction, including changes caused by alterations in the mechanic field parameters.

## Mechanic and gravitational sensitivity in different types of bone tissue cells: effects on osteogenic cell differentiation.

Studies that focus on the various parameters of collagen biosynthesis of the so–called mechanocytes (fore mostly fibroblasts and bone cells) under conditions of elevated or decreased gravity are of especial interest. Hypergravity usually results in increased type I collagen biosynthesis [54], while microgravity or their modeling suppress the expression of this protein [4, 55]. Our study found that MMSC, which were committed to osteogenesis simultaneously with the transfer of cells into simulated microgravity, displayed a decrease in the production rate of extracellular collagen matrix (type I collagen) [56].

A correlation has been found between the level of collagen synthesis and the activation of MAPK–family kinases, and ERK1/2 in particular, since inhibition of this signaling pathway caused decreased gene expression levels and decreased protein production levels of one of the chains of type I collagen [54]. MMSC cells committed to osteogenesis displayed a complete lack of type I collagen expression coupled with changes in the expression levels of integrins, specific to collagen, after 7 days of cultivation in a rotational bioreactor. They also exhibited decreased levels of ERK1/2^MAPK^ phosphorylation, as opposed to p38^MAPK^ phosphorylation levels, which were elevated [37, 49].

**Fig. 1:**
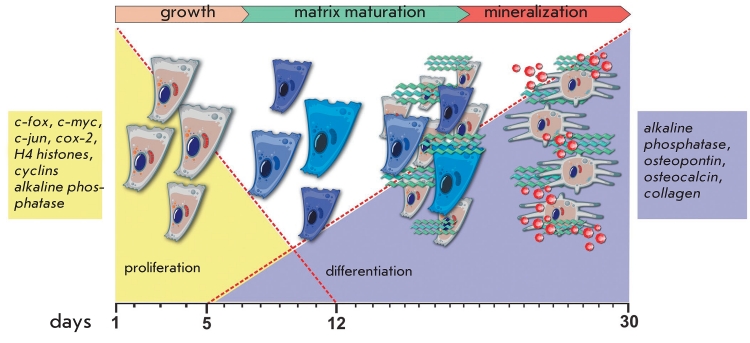
Relationships between growth and differentiation stages of osteoblasts. Early stages of osteogenic cell growth are regulated by early response genes *c-fos*, *c-myc*, *cox-2* and *efr-1*, as well as by transcription factors which are activated in the cells that have commenced their cell cycle. During the very late proliferation stage and early differentiation stage, alkaline phosphatase, collagen, and fibronectin are upregulated. In the middle of the matrix maturation stage, mineralization genes and alkaline phosphatase are activated. Adapted from [5]

It was shown that during the course of induced osteogenesis in normal cells of osteoblastic phenotype the activation of type I collagen expression begins after 5–6 days of cell cultivation in osteogenic medium, and the peak of protein expression was usually reached on days 9–14, which was the end of the proliferative phase and the beginning of the so–called matrix maturation period [57]. This indicated that a short–term exposure of cells to microgravity did not necessarily cause expression inhibition for relatively “late” phenotypic genes, such as collagen. This also probably means that the expression of any “mechanically sensitive” osteoblast protein product is most vulnerable to changes in the gravitational field at the peak of its expression, which is tightly connected with three distinct differentiation phases in cells of osteoblastic phenotype (Fig. 1).

Another important aspect of the effects of microgravity on osteogenic cell differentiation is the decrease of expression and activity levels of the alkaline phosphatase (APL), and the expression inhibition of the late mineralized bone matrix marker proteins, such as osteopontin and osteocalcin, which indicate slowing effects on both the early and the late phases of osteogenic precursor cells’ differentiation into osteoblasts [4, 36, 37, 49, 55, 58]. APL definitely plays a role in the mineralization of the bone matrix in bone tissue; nevertheless, it is still unclear how this mechanism functions, and the precise function of the enzyme remains an issue for discussion [59]. It is not always possible to see a direct correlation between the activity of this enzyme and certain observed physiological effects, which indicates that other pathways of mineralized matrix formation may exist. One study, in particular, demonstrates that the mechanic stimulation of osteoblasts by pulsatile fluid flow causes an increase in the cellular activity of APL, although it is not accompanied by an increase of matrix mineralization in the culture [60]. One possible explanation might be provided by the recently proposed hypothesis stating that the cells of the osteocyte lineage play a role in the formation of a stable morphologically structured bone matrix. The authors propose that depending on external factors bone cells regulate the formation, maturation, and rate of crystallization of amorphous phosphate–calcium mineralization nuclei via non–collagenous proteins of the bone tissue (osteonectin, osteopontin, osteocalcin and bone sialoprotein) [61]. Interestingly, lowered expression of * osteocalcin * in cells under micrigravity is often accompanied by lowered expression of a key transcription factor, which regulates osteogenic differentiation of osteogenic cells. This factor is Runx2 (runt–related transcription factor 2), and it may be one of the primary “targets” of microgravitational effects on the osteoblastic phenotype.

## The role of Runx2 in the regulation of osteogenic differentiation of MMSC and osteoblasts and its potential role as the main “target” of altered gravity effects

Runx2/PEBP2aA/Cbfa1, the main regulator of mesenchymal cell osteogenic differentiation, which can respond to the effect of osteogenic growth factors, was first identified in the course of studies connected with osteogenic differentiation of pluripotent mesenchymal precursor–cells of the C2C12 mouse line [62]. Full–fledged osteoblast differentiation and expression of specific osteogenic genes requires the cooperation of Runx2 and Smad molecules, which are activated by BMP–2. It was also discovered that growth factor BMP–7 induced the expression of Runx2 mRNA earliear than osteocalcin expression, and furthermore, transfection by an isoform of *Runx2 * led to the osteogenic differentiation of non–osteogenic cells [63].

It is currently accepted that Runx2 is an essential, but not the only, needed, osteogenesis transcription factor. It cooperates in the postnatal development of osteoblasts with other transcription factors (Osx, Msx, Smad, Dlx) and also plays a key role in the regulation of osteogenic differentiation of mesenchymal cells [64, 65]. Preosteoblasts which experience mechanical deformation respond with rapid activation of * BMP–2, Runx2* *и  Smad5 * expression, and this effect is later followed by increases of the expression of genes needed for the formation and maturation of the matrix: *ALP, COL1a1 * and * OC, OPN * [66]. “Mechanically sensitive” genes were identified in osteoblasts under conditions of simulated microgravity, with * Runx2 * being one of them [67]. It was also demonstrated that LMHF (low magnitude and high frequency mechanical loading) of preosteoblasts could prevent the suppression of the osteogenic differentiation potential of cells under simulated microgravity. This was accompanied by restoration of the previously suppressed expression of * Runx2* [68]. Notably, * in vivo * models also showed that deactivation or lowering of the expression level of * Runx2 * were among the main mechanisms by which hypokinesia affected the osteoblastic phenotype. Partial *Runx2 * heterozygous knockout mice were particularly sensitive to unloading, which provoked a more noticeable loss of bone tissue mass than in wild–type mice with a normal level of * Runx2 * expression [69].

## The role of mechanical signals in determining the differentiation fate of mesenchymal stromal precursor cells: PPAR γ 2 versus Runx2

The organism possess a highly surprising connection between osteogenesis and adipogenesis, which is preserved in the cultured precursor cells as well. Probably, these unusual reciprocal interactions between the two differentiation lineages of MMSC are determined by shared signaling pathways and regulating mechanisms, which prioritize the development of one differentiation path at the expense the other one, basing this choice on the signals received by the cells. At least some of these mechanisms have recently been elucidated.

PPAR γ 2 is a key adipogenic transcription factor and it functions as a negative dominant regulator of osteogenesis [70]. Specific activation of PPAR γ 2 by various natural and synthetic ligands leads to complete suppression of the main transcription factors of osteogenesis, cbfa1/Runx2 and Osterix, and also to increased conversion of bipotent mesenchymal precursors into adipocytes, without affecting the morphofunctional condition of osteoblasts, which are in the terminal stages of differentiation [71]. Chronic injection of a PPAR γ 2 antagonist rosiglitazone leads to a loss of bone tissue mass in mice, which is accompanied by an increase in the number of adiopcytes in the murine bone tissue and a decrease in the osteoblast to osteoclast ratio [72, 73]. Interestingly, as MMSC age, the activity of PPAR γ 2 increases, which correlates with the decrease of the pool of osteoblasts and the elevation of adipocytes numbers in the bone marrow. The cells also experience lowered expression levels of * Runx2 * и * Dlx5* , and also decreased production of collagen and osteocalcin [74].

Recently, the role of mechanical signals in determining and realizing various MMSC differentiation programs has been a highly discussed topic [75]. It was determined that mechanical stretching led to lowered PPAR γ 2 levels in a culture of bovine MMSC and in a C3H10T1/2 cell line [76]. It was also shown that mechanical stimuli led to elevated expression levels of Msx2, which activated osteogenic differentiation of cells, showed a synergistic effect with BMP–2 and inhibited PPAR γ 2, thus acting as a suppressor of adipogenesis [77]. Transitory activation of the Wnt/ β –Catenin signaling pathway inhibited adipogenic differentiation of cells by suppressing * C/EBP* *α * and *PPAR* *γ* *2 * expression and activating the expression of osteogenetic transcription factors * Runx2, Dlx5 * and *Osterix * [78]. Other studies demonstrated the possibility of the Wnt/ β –Catenin–signalling pathway being implicated in the inhibition of adipogenesis and stimulation of cell osteogenesis in response to mechanical deformation. This process was realized via estrogen α –receptors [79] and insensitive to the powerful adipogenesis inducers which were present in the cell culture media [80]. Interestingly, mouse osteoblasts subjected to simulated microgravity were found to have suppressed levels of several components of the Wnt/ β –Catenin–signalling pathway, such as * Sfrp2 * and * Wisp2, * which may indicate, albeit indirectly, the activation of an adipogenic program under microgravity [67]. It was also shown that MMSC extracted from the bone marrow of unloaded rats and cultured * ex vivo * exhibited lowered levels of *cbfa1/Runx2 * expression during the activation of osteogenic differentiation. On the other hand, these cells demonstrated an increased expression of * PPAR* *γ* *2 * during activation of adipogenic differentiation and generally differentiated more easily into the adipogenic lineage [81]. Similar changes were seen after short incubations of MMSC in a rotational reactor, which models the effects of microgravity [37]. However, studies of induced adipogenic differentiation of MMSC under prolonged incubations in simulated microgravity did not yield any phenotypic signs of increased adipogenesis in MMSC [56].

**Fig. 2:**
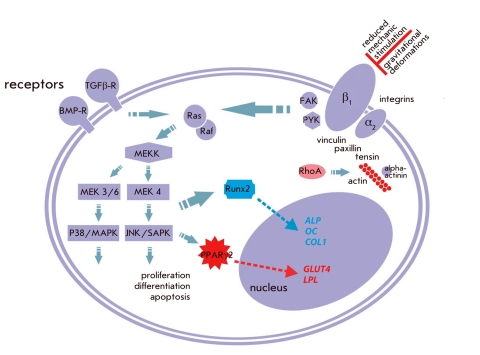
Molecular regulation of the proliferation and differentiation of MMSCs under the control of an extracellular mechanical field. Extracellular signals and mechanical stimuli or their absence act via putative extracellular channels and receptors (e.g., integrins) and possibly trough other still unknown mechanisms. Signals are transferred from integrins to integrin-related focal adhesion kinases (FAK, PYK), which are, in turn, involved in multiple signal transduction pathways, including MAP-kinases, cellular cytoskeleton effectors (vinculin, paxillin, and talin), and Rho-kinases (ROCK). MAP–kinases regulate the main cell processes, such as proliferation, differentiation, and apoptosis. Rho-kinases perform actin cytoskeleton remodeling. Вlue arrows indicate Runx2 signals to the nucleus; orange arrows indicate PPARγ2 signals

Microgravity can modify the differentiation potential of precursor cells through changes in the activity of the major kinase signal transduction cascades (Fig. 2). It was determined that MAP–kinases played an important, if not a key role, in regulation of the differentiation potential of mesenchymal origin cells, including the cells under mechanical stress conditions [41, 82, 83]. For instance, it was shown that phosphorylation of Runx2 by MAP–kinases was needed in order for this protein to function in transcription activation [84]. Also, decreased/altered levels of MAP–kinase activity were an often seen cell response under simulated gravity conditions. Lowered levels of phosphorylated ERK1/2^MAPK^ during the process of MMSC osteogenic differentiation in a rotational bioreactor [37, 38] or decreased levels of phosphorylated p38 ^МАРК ^ during osteogenic ostoeblast differentiation in a 3D–clinostat [58] have also been reported.

## The role of autocrine signals in the regulation of the morpho-functional state and the commitment of mesenchymal stromal precursor-cells under conditions of microgravity

The reciprocal suppression of the two differentiation pathways of MMSC may be attributed to the existence of other regulatory mechanisms, including those of autocrine and paracrine nature. For instance, the products of one of the differentiation pathways may inhibit the production of compunds which are needed for the formation of the other phenotype. Studies have shown that the lipoprotein lipase produced by adipocytes could bind sortilin, the expression of which was induced during osteogenic differentiation of MMSC, since this receptor protein was needed for the normal mineralization of the bone matrix. Moreover, sortilin itself was able to mediate the endocytosis of lipoprotein lipase [85]. It has also been shown that the increase in adipogenic differentiation of MMSC’s obtained from osteoporosis patients was caused by an abnormal response of the cells to the leptin cytokine, which usually suppressed PPAR γ by phosphorylation [86].

The functional role of most cytokines in the regulation of the MMSC lifecycle and in the adaptation of these and other osteogenic cells to microgravity has not been studied very deeply and requires further investigations. During recent years, researchers have paid much attention to the role of IL–8. It is known that the expression of the neutrophil–activating factor is regulated by IL–1 β and TNF–a, and also by glucocorticoid hormones. Notably, IL–8 can regulate the expression of cell adhesion molecules, and also the excretion of several enzymes which can degrade the extracellular matrix [87]. These properties of IL–8 can be important for the local mechanisms of bone tissue remodeling, which are a part of several local cellular responses to microgravity. For example, bioptates of Macaca mulatta bones exposed to microgravity on the bio–sattelite “Bion–11” were found to exhibit activated resorption mediated by osteoclast resorption and osteocyte osteolysis. Neturophile activity was also elevated, and these cells excreted hydrolytic enzymes, which took part in the destruction of the mineralized bone matrix [88]. It was recently shown that the production of IL–8 in MMSC increased in response to repeated mechanic stretching; moreover, cells cultured in osteogenic medium showed the highest increase of IL–8 production [89]. Interestingly, a manifold activation of IL–6 and IL–8 production was seen in endothelial cells, which were subjected to simulated microgravity, using an RPM [90]. It was shown that the cells exhibiting different levels of commitment (MMSC and their derivative osteogenic cells, and also osteoblasts) all responded to prolonged incubations in simulated microgravity in a similar manner, by an increased level of autocrine IL–8 production [91].

## The role of the cytoskeleton in gravitational sensitivity of MMSC under altered gravity

**Fig. 3:**
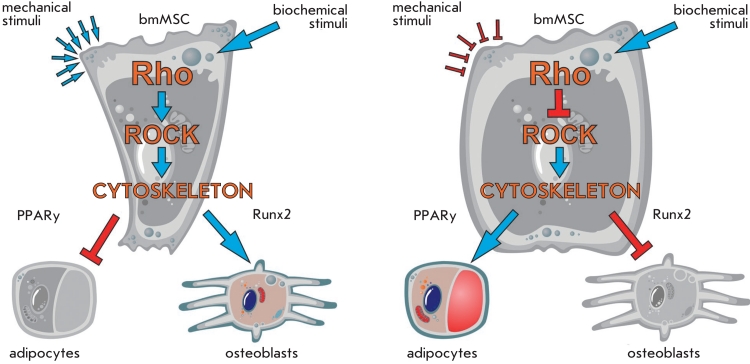
Model of a mechanically mediated switch in MSC commitment. The cell shape acts as a mechanical cue, driving MSC commitment between adipocyte and osteoblast when RhoA signaling and cytoskeletal tension are intact. Well-spread cells prefer to differentiate into the osteogenic lineage and round cells prefer to differentiate into the adipogenic lineage. When the cell shape is changing active RhoA is sufficient to replace biochemical stimuli whereas RhoA effector - ROCK acts independently of cell shape. Interference with the cell shape, RhoA signaling, ROCK activity, or cytoskeletal tension alters hMSC commitment. Red arrows indicate blocking of differentiation, blue arrows – activation of differentiation

Recently, there are more and more observations giving strength to the idea that cytoskeletal structures and cell surface receptors connected to them play an imporatant role in the regulation of the differentiation potential of stem cells, which is affected by signals from an “external mechanical field” (Fig. 3). Also, changes of shape and of the inner cytoskeletal architecture are common cell responses under conditions of real [22] or simulated microgravity [26, 46, 92]. It has been determined that changes in the morphological characteristics of cells, or modulation of the Rho family proteins activity (GTPases that regulate actin cytoskeleton) can lead to the modification of the differentiation potential of MMSCs. For instance, activation of Rho–kinase (ROCK) by the upstream RhoA GTPase can induce the myogenic MMSC differentiation pathway and inhibit the adipogenic pathway even in the presence of the insulin–like IGF–I factor [93]. It is proposed that cell shape can act as a mechanical stimulus and plays an important role in the determination of the differentiation pathway of the precursor cells. It was shown that well spread cells were inclined to differentiate down the osteogenic pathway, while round unspread cells tended to take the adipogenic fate. Expression of the dominant negative * RhoA* caused differentiation into adipocytes, while overexpression of the wild–type gene led to osteogenesis. The authors found that normal actin–myosin tension was required for the correct activation of Rho–kinases by RhoA and suggested that the cytoskeleton and the regulatory proteins coupled to it could act as an integral regulatory system that controlled cell differentiation decisions, which were mainly defined through mechanical signals [94]. Interestingly, cultivation of MMSC under simulated microgravity caused changes in the actin cytoskeleton, up to a complete absence of filamentous actin in the cell after a 7–day incubation. Another effect was a strong drop in the activity of RhoA–kinase. Moreover, transfection of the cells by a viral vector, which expressed a constitutively active * RhoA * , prevented the described cytoskeleton alterations and neutralized the development of adipogenic features in the cells [92]. Direct interaction between ERK1/2^MAPK ^with the integrin–mediated signaling pathway and also with the activity of several cytoskeletal effector proteins was demonstrated by switching–off of one of the actin cytoskeleton remodeling proteins (Rho), which caused the inactivation of the MAP–kinase cascade [95].

Studying all the complex factors that control the commitment of MMSC cell differentiation can help elucidate the mechanisms which are required for maintaining the delicate equilibrium between the two stem cell differentiation pathways. Deregulation of this equilibrium during hypokinesia or microgravity can lead to severe medical conditions, such as osteopenia or osteoporosis. In conclusion, it is worth repeating that multipotent mesenchymal stromal cells of the human bone marrow are the population of cells with low–level commitment which are sensitive to gravitational changes. Despite the growing number of reports on the effect of real or microgravity on the morpho–functional state of various types of cultured osteogenic cells, the precise molecular and intracellular mechanisms of the observed effects are still not fully understood. However, the overall phenomenology of responses from osteogenic cells of various levels of commitment indicates that common mechanisms for sensing and responding to alterations in the gravitation field do exist. Further comprehensive studies in this field will facilitate fundamental understanding of the mechanisms of gravitational and mechanical sensitivity of adult precursor cells and their possible involvement in the local cell reactions, which take place in the bone tissue in microgravity.

## REFERENCES

1Grigoriev A.I.Egorov A.D. Human in space flightSpace biology and medicineNaukaMoscow199435495492Oganov V.S. SlovoMoscow20032602603Akiyama H.Kanai S.Hirano M. Mol.Cell Biochem.19992021637110.1023/a:1007097511914107059964Carmeliet G.Nys G.Bouillon R. J. Bone Miner Res.1997125786794914434510.1359/jbmr.1997.12.5.7865Hughes-Fulford M. Cell biology and biotechnology in space Ed by A Cogoli.Advances in space biology and medicine200281291571295169510.1016/s1569-2574(02)08017-66Hughes-Fulford M.Rodenacker K.Jütting U. J. Cell Biochem.20069924354491661926710.1002/jcb.208837Kumei Y.Morita S.Katano H. Ann. NY Acad. Sci.200610903113171738427510.1196/annals.1378.0348Kumei Y.Shimokawa H.Ohya K. Ann. NY Acad. Sci.200710952922991740404110.1196/annals.1397.0329Bianco P.Riminucci M.Gronthos S.Robey P.G. Stem Cells20011931801921135994310.1634/stemcells.19-3-18010Crisan M.Yap S.Casteilla L. Cell Stem. Cell20083330131310.1016/j.stem.2008.07.0031878641711Majumdar M.K.Keane-Moore M.Buyaner D. J. Biomed. Sci.20031022282411259575910.1007/BF0225605812Pittenger M.F.Mackay A.M.Beck S.C. Science199928454111431471010281410.1126/science.284.5411.14313Wagner W.Roderburg C.Wein F. Stem Cells20072510263826471761526210.1634/stemcells.2007-028014Burger E.H.Klein-Nulend J. Bone199822512712710.1016/s8756-3282(98)00010-6960076815Mikuni-Takagaki Y.Suzuki Y.Kawase T.Saito S. Endocrinology1996137520282035861254410.1210/endo.137.5.861254416Parfenov G.P. Ugolev A.M.Problems kosm.Biol.NaukaLeningrad198857667717Tairbekov M.G. Dokl. Biol Sci.2000375112112410.1023/a:10266227286991121151918Lambert C.A.Lapiere C.M.Nusgens B.V. Brinckmann E.The gravity environment in space flight. Biology in Space and life on Earth.Effects of spaceflight on biological systemsWiley-VCH Verlag GmbH & Co. 200712315519Moore D.Cogoli A. Moore D. Oser H. Gravitational and space biology at the cellular level. Biological and medical research in space.An overview of life sciences research in microgravitySpringerHamburg1996110720van Loon J.J.W.A. BrinckmannE.The gravity environment in space flight. Biology in Space and life on Earth.Effects of spaceflight on biological systemsWiley-VCH Verlag GmbH & Co.2007173221Buravkova L.B. Aviakosm. Ekolog. Med.200842610181923891322Lewis M. Cell biology and biotechnology in space.Advances in space biology and medicine20028771281295169410.1016/s1569-2574(02)08016-423Ingber D.E. Proc. Natl. Acad. Sci. USA20031004147214721257896510.1073/pnas.0530201100PMC14985424Ingber D.E. Tensegrity I. J. Cell Sci.2003116115711731261596010.1242/jcs.0035925Albrecht-Buehler G. ASGSB Bull.19914225341153717926Wang Y.McNamara L.M.Schaffler M.B.Weinbaum S. Proc. Natl. Acad. Sci. USA20071044015941159461789537710.1073/pnas.0707246104PMC200040527Ajubi N.E.Klein-Nulend J.Alblas M.J. Am. J. Physiol.1999276117117810.1152/ajpendo.1999.276.1.E171988696428Pitsillides A.A.Rawlinson S.C.Suswillo R.F. FASEB J.199591516141622852984110.1096/fasebj.9.15.852984129McGarry J.G.Klein-Nulend J.Mullender M.G.Prendergast P.J. FASEB J.20051934824841562508010.1096/fj.04-2210fje30Smalt R.Mitchell F.T.Howard R.L.Chambers T.J. Am. J. Physiol. Endocrinol. Metab. 199727375175831Searby N.D.Steele C.R.Globus R.K. Am. J. Physiol. Cell Physiol.2005289114815810.1152/ajpcell.00524.20031572871032Arikawa T.Omura K.Morita I. J. Cell Physiol200420034004061525496810.1002/jcp.2003133Kurpinski K.Chu J.Hashi C.Li S. Proc. Natl. Acad. Sci. USA20061034416095161001706064110.1073/pnas.0604182103PMC163754234Rucci N.Migliaccio S.Zani B.M. J. Cell Biochem.20028511671791189186035Sato A.Hamazaki T.Oomura T. Adv. Space Res.19992468078131154262610.1016/s0273-1177(99)00076-936Pardo S.J.Patel M.J.Sykes M.C. Am. J. Physiol. Cell Physiol.200528861211122110.1152/ajpcell.00222.20041568941537Zayzafoon M.Gathings W.E.McDonald J.M. Endocrinology20041455242124321474935210.1210/en.2003-115638Dai Z.Q.Wang R.Ling S.K. Cell Prolif.20074056716711787760910.1111/j.1365-2184.2007.00461.xPMC649637139Yuge L.Kajiume T.Tahara H. Stem Cells Dev.20061569219291725395310.1089/scd.2006.15.92140Boonstra J. FASEB J.199913354210.1096/fasebj.13.9001.s351035214341Jaiswal R.K.Jaiswal N.Bruder S.P. J. Biol. Chem.200027513964596521073411610.1074/jbc.275.13.964542CelilA.B.CampbellP.G.J. Biol. Chem.20052803631353313591600030310.1074/jbc.M50384520043RiddleR.C.TaylorA.F.GenetosD.C.DonahueH.J.Am. J. Physiol. Cell. Physiol.2006290377678410.1152/ajpcell.00082.20051626710944CongetP.A.MinguellJ.J.J. Cell. Physiol.1999181167731045735410.1002/(SICI)1097-4652(199910)181:1<67::AID-JCP7>3.0.CO;2-C45BuravkovaL.B.MerzlikinaN.V.RomanovY.A.BuravkovS.V.J. Grav. Physiol.200512124124246GershovichP.M.GershovichJ.G.BuravkovaL.B.Cell and Tissue Biol.20093542343047MiyamotoS.TeramotoH.CosoO.A.J. Cell Biol.19951313791805759319710.1083/jcb.131.3.791PMC212062048IwaniecU.T.WronskiT.J.AmblardD.J. Appl. Physiol.20059826906961546588810.1152/japplphysiol.00689.200449MeyersV.E.ZayzafoonM.GondaS.R.J. Cell Biochem.20049346977071566041410.1002/jcb.2022950YouJ.ReillyG.C.ZhenX.J. Biol. Chem.20012761613365133711127857310.1074/jbc.M00984620051IshijimaM.TsujiK.RittlingS.R.J. Endocrinol.200719322352431747051410.1677/joe.1.0670452OhnoS.ImH.J.KnudsonC.B.KnudsonW.J. Biol. Chem.20062812617952179601664863310.1074/jbc.M602750200PMC313922953KasperG.GlaeserJ.D.GeisslerS.Stem Cells.2007258198519941749511310.1634/stemcells.2006-067654GebkenJ.LüdersB.NotbohmH.J. Biochem.199912646766821050267410.1093/oxfordjournals.jbchem.a02250255NarayananR.SmithC.L.WeigelN.L.Bone20023133813881223141010.1016/s8756-3282(02)00836-056GershovichJ.G.GershovichP.M.BuravkovaL.B.Technologies of life system200923957SteinG.S.LianJ.B.Endocr. Rev.1993144424442822334010.1210/edrv-14-4-42458YugeL.HideI.KumagaiT.In Vitro CellDev. Biol. Anim.2003391899710.1290/1543-706x(2003)039<0089:cdapca>2.0.co;21289253259SugawaraY.SuzukiK.KoshikawaM.Jpn. J. Pharmacol.20028832622691194988010.1254/jjp.88.26260NaumanE.A.SatcherR.L.KeavenyT.M.J. Appl Physiol.2001905184918541129927610.1152/jappl.2001.90.5.184961AvruninA.S.KornilovN.V.MarinU.B.Morfologiia2002674771263010262Lee K-S.Kim H-J.Li Q.-L.Mol. аnd Cell. Biol.200020238783879210.1128/mcb.20.23.8783-8792.2000PMC865111107397963DucyP.SchinkeT.KarsentyG.Science20002895484150115041096877910.1126/science.289.5484.150164Lee M-H.Kim Y-H.Kim H-J.J. Biol. Chem.20032783634387343941281505410.1074/jbc.M21138620065YamaguchiA.KomoriT.SudaT.Endocr. Rev.20002143934111095015810.1210/edrv.21.4.040366RathB.NamJ.KnoblochT.J.J. Biomech.2008415109511031819113710.1016/j.jbiomech.2007.11.024PMC229154767CapulliM.RufoA.TetiA.RucciN.Journal of Cellular Biochemistry2009102402521928852710.1002/jcb.2212068PatelM.J.ChangK.H.SykesM.C.J. Cell Biochem.200910623063161912541510.1002/jcb.22007PMC273772169SalingcarnboriboonR.TsujiK.KomoriT.Endocrinology20061475229623051645578010.1210/en.2005-102070ShockleyK.R.RosenC.J.ChurchillG.A.Lecka-CzernikB.PPAR Res.2007200781219812191828826610.1155/2007/81219PMC223408871Lecka-CzernikB.MoermanE.J.GrantD.F.Endocrinology20021436237623841202120310.1210/endo.143.6.883472AliA.A.WeinsteinR.S.StewartS.A.Endocrinology20051463122612351559115310.1210/en.2004-073573LazarenkoO.P.RzoncaS.O.HogueW.R.Endocrinology20071486266926801733206410.1210/en.2006-1587PMC208445974MoermanE.J.TengK.LipschitzD.A.Lecka-CzernikB.Aging Cell.2004363793891556935510.1111/j.1474-9728.2004.00127.xPMC185010175EstesB.T.GimbleJ.M.GuilakF.Curr. Top. Dev. Biol.200460911261509429710.1016/S0070-2153(04)60004-476DavidV.MartinA.Lafage-ProustM.H.Endocrinology20071485255325621731777110.1210/en.2006-170477ChengS.L.ShaoJ.S.Charlton-KachigianN.J. Biol. Chem.20032784645969459771292552910.1074/jbc.M30697220078KangS.BennettC.N.GerinI.J. Biol Chem.20072821914515145241735129610.1074/jbc.M70003020079ArmstrongV.J.MuzylakM.SuntersA.J. Biol. Chem.20072822820715207271749102410.1074/jbc.M70322420080SenB.XieZ.CaseN.Endocrinology200814912606560751868777910.1210/en.2008-0687PMC261306881PanZ.YangJ.GuoC.Stem Cells Dev.20081747958041871034610.1089/scd.2007.025482BoutaharN.GuignandonA.VicoL.Lafage-ProustM.H.J. Biol. Chem.20042792930588305991509650210.1074/jbc.M31324420083SalasznykR.M.KleesR.F.WilliamsW.A.Exp. Cell Res.2007313122371708151710.1016/j.yexcr.2006.09.013PMC178017484XiaoG.JiangD.ThomasP.J. Biol. Chem.20002756445344591066061810.1074/jbc.275.6.445385MaedaS.NobukuniT.Shimo-OnodaK.J. Cell Physiol.2002193173791220988210.1002/jcp.1015186AstudilloP.RíosS.PastenesL.J. Cell Biochem.20081034105410651797327110.1002/jcb.2151687ChaudharyL.R.AvioliL.V.J. Biol. Chem.1996271281659116596866317910.1074/jbc.271.28.1659188RodionovaN.VOganovV.S.J .Gravit. Physiol.20018187881265018689SumanasingheR.D.PfeilerT.W.Monteiro-RiviereN.A.LoboaE.G.J. Cell Physiol.2009219177831908999210.1002/jcp.2165390UlbrichC.WestphalK.BaatoutS.J. Cell. Biochem.20081044132413411825393610.1002/jcb.2171091GershovichJ.G.BuravkovaL.B.Aviakosm. Ekolog. Med.2009344501971186292MeyersV.E.ZayzafoonM.DouglasJ.T.McDonaldJ.M.J. Bone Miner. Res.20052010185818661616074410.1359/JBMR.050611PMC135102093SordellaR.JiangW.ChenG.C.Cell200311321471581270586410.1016/s0092-8674(03)00271-x94McBeathR.PironeD.M.NelsonC.M.Dev. Cell.2004644834951506878910.1016/s1534-5807(04)00075-995RenshawM.W.ToksozD.SchwartzM.A.J. Biol. Chem.1996271362169121694870296010.1074/jbc.271.36.21691
